# Supplementary material for: In Vivo-Matured Oocyte Resists Post-Ovulatory Aging through the Hub Genes DDX18 and DNAJC7 in Pigs
Source: Antioxidants (Basel). 2024 Jul 19;13(7):867. doi: 10.3390/antiox13070867 (PMC11274268; doi:10.3390/antiox13070867)
Supplement: Supplementary file 1 [file antioxidants-13-00867-s001.zip › antioxidants-2961772-supplementary.pdf]

Supplementary Material

1. RNA sequencing data analysis

1.1 Clean Reads Filtering

To get high-quality clean reads, reads were filtered by fastp [1] (version 0.18.0) to remove reads that contain adapters, more than 10% of unknown nucleotides(N), and more than 50% of low-quality (Q-value≤20) bases.

1.2 Ribosome RNA (rRNA) Alignment

Short reads were aligned to the rRNA database using Bowtie2 [2] (v2.2.8). The resulting rRNA-mapped reads were removed, and the remaining clean reads were used for gene assembly and abundance calculation.

1.3 Alignment with Reference Genome

An index of the reference genome was built, and paired-end clean reads were mapped to the reference genome using HISAT2.4 [3] and other parameters set as a default. The reference genome used in this study is ncbi_GCF_000003025.6.

1.4 Gene Abundance Calculation

Use the StringTie [4] (v 1.3.1) to assemble the mapped reads of every sample in a reference-based approach. For each transcript, the expression abundance and variations were quantified by calculating a fragment per kilobase of transcript per million mapped reads (FPKM) value using RSEM [5] software.

The FPKM formula is as follows:

$$FPKM = \frac{10\text{^}6C}{NL/10\text{^}3}$$

Given FPKM(i) as the expression of gene i, C as the number of fragments mapped to gene i, N as the total number of fragments mapped to reference genes, and L as the number of bases on gene i.

1.5 Relationship analysis of samples

1.5.1 Correlation Analysis of Replicas

R (v 4.3.2) was used to perform correlation analysis on two parallel experiments. The correlation coefficient was calculated between two replicas to determine the repeatability between samples.

1.5.2 Principal Component Analysis

Principal component analysis (PCA) was performed with R package models (http://www.r-project.org/) in this experience.

1.6 Differentially expressed genes (DEGs) Analysis

RNAs differential expression analysis was performed by DESeq2 [6] software between two different groups. The genes/transcripts with the parameter of false discovery rate (FDR) below 0.05 and absolute fold change≥2 were considered differentially expressed genes/transcripts.

1.6.1 Gene Ontology (GO) Enrichment Analysis

All DEGs were assigned GO terms from the GO database (http://www.geneontology.org/). The number of genes was calculated for each term, and significantly enriched GO terms in DEGs compared to the genome background were identified using a hypergeometric test. The calculating formula of the P-value is as follows:

$$P = 1 - \sum_{i=0}^{m-1} \frac{\binom{M}{i}\binom{N-M}{n-i}}{\binom{N}{n}}$$

N represents the total number of genes with GO annotation, n represents the number of DEGs in N, M represents the total number of genes that are annotated to specific GO terms, and m represents the number of DEGs in M. The obtained p-values underwent FDR Correction, where a threshold of FDR ≤ 0.05 was considered significant. GO terms that met this condition were defined as significantly enriched in DEGs.

1.6.2 Pathway Enrichment Analysis

The Kyoto Encyclopedia of Genes and Genomes (KEGG) (https://www.genome.jp/kegg/) was used to map all DEGs to pathways. Gene numbers were determined for each pathway, and a hypergeometric test was conducted to identify significantly enriched KEGG pathways in DEGs compared to the genome background. The calculation formula used in GO analysis was also applied here.

1.7 Protein-Protein interaction

The String v10 tool was used to identify a Protein-Protein interaction network. Genes were nodes and interactions were lines in a network. Cytoscape (v3.1.1) was used to visualize the network and identify core and hub genes.

1.8 Weighted Gene Co-Expression Network Analysis

The Weighted Gene Co-Expression Network Analysis (WGCNA) package [7] screened hub genes that were significantly associated with POA. Take all genes with FPKM > 2 in all groups (Total of 11,804 genes) (Table S2) as the input. The applicable power value for this test was nine (Table S3). Then, gene modules were detected based on the TOM matrix. The most positive correlation was selected for further analysis through the calculation of the Pearson correlation coefficient between the modules and traits. Gene significance (GS) for traits of each gene and module membership (MM) in the hub module was then measured. Genes in the module were screened for potential CAF-related characteristics using thresholds of MM >0.6 and GS >0.6.

2. Supplementary Figures

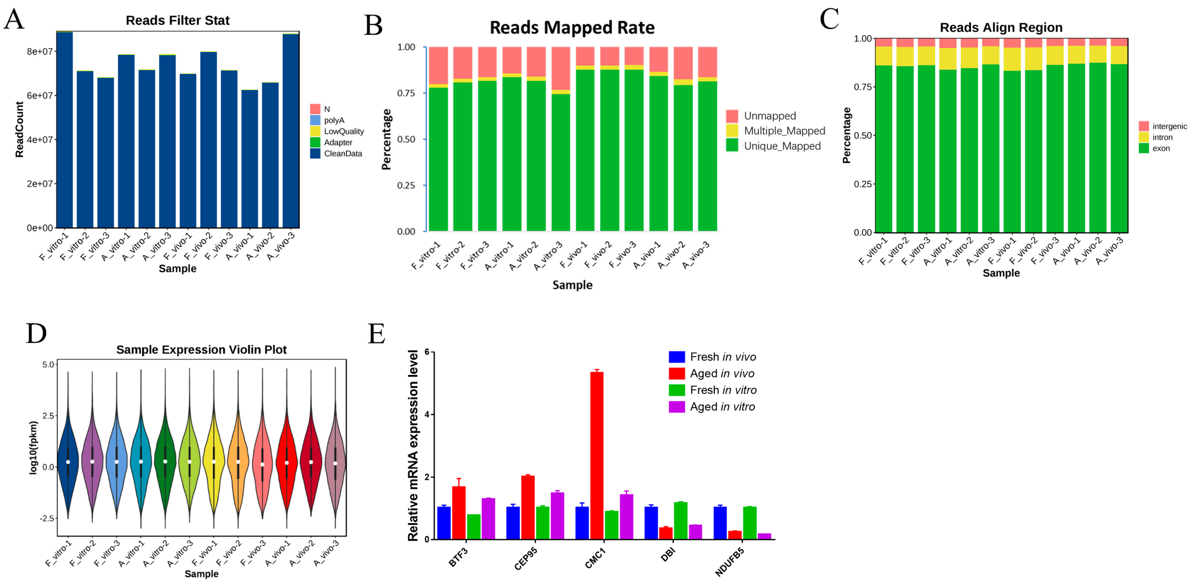

Figure S1. Filter, Mapping, alignment, and verification of RNA sequencing data. (A) Reads filter statistical chart (numerical); sequencing obtained a total of 62.7–89.2 million raw reads, which were filtered to obtain 62.4–88.6 million clean reads. (B) Reads mapped rate statistical chart; 76.77–90.21 % of the clean reads were mapped to the reference genome. (C) Read align region statistical chart; 83.28–87.44 % were mapped to the exon region. (D) Violin plot of gene expression; total gene expression of all samples is uniform and concentrated. (E) Relative mRNA expression levels of *BTF3*, *CEP95*, *CMC1*, *DBI*, and *NOUFB5* in four groups.

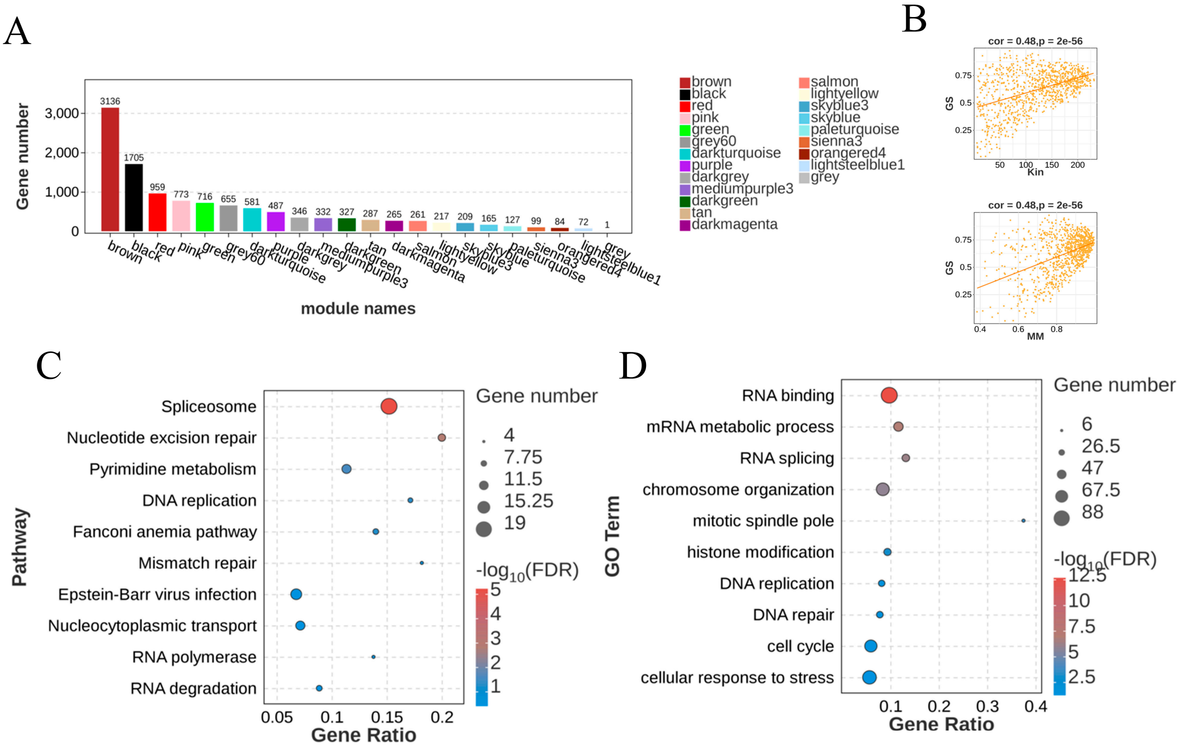

Figure S2. Correlation analysis and functional enrichment of gene modules specifically expressed in *in vivo* matured aging oocytes. (A)The bar plot shows the number of genes in every module. (B) The (module membership) MM- gene significance (GS) correlation of genes in the red module. Use the GS and MM values of genes to analyze the correlation between each trait and the module. Modules with

high correlation play an important biological role in the trait. Intramodular connectivity (K.in) and GS correlation analysis of genes in the red module. Analyze the association between modules, genes, and traits using the connectivity of genes within the module (K.in value) and the correlation value between genes and traits (GS). (C) KEGG enrichment analysis of genes in the red module showing the top 15 enriched KEGG pathways. (D) GO enrichment analysis of genes in the red module showing the top 15 enriched GO terms.

[1] S. Chen, Y. Zhou, Y. Chen, J. Gu, fastp: an ultra-fast all-in-one FASTQ preprocessor, Bioinformatics 34(17) (2018) i884-i890.
[2] B. Langmead, S.L. Salzberg, Fast gapped-read alignment with Bowtie 2, Nat Methods 9(4) (2012) 357-9.
[3] D. Kim, B. Langmead, S.L. Salzberg, HISAT: a fast spliced aligner with low memory requirements, Nat Methods 12(4) (2015) 357-60.
[4] M. Pertea, G.M. Pertea, C.M. Antonescu, T.C. Chang, J.T. Mendell, S.L. Salzberg, StringTie enables improved reconstruction of a transcriptome from RNA-seq reads, Nat Biotechnol 33(3) (2015) 290-5.
[5] B. Li, C.N. Dewey, RSEM: accurate transcript quantification from RNA-Seq data with or without a reference genome, BMC Bioinformatics 12 (2011) 323.
[6] M.I. Love, W. Huber, S. Anders, Moderated estimation of fold change and dispersion for RNA-seq data with DESeq2, Genome Biol 15(12) (2014) 550.
[7] P. Langfelder, S. Horvath, WGCNA: an R package for weighted correlation network analysis, BMC Bioinformatics 9 (2008) 559.
